# The Treatment of Runner’s Dystonia With Zolpidem and a Device Inducing Hanger Reflex

**DOI:** 10.7759/cureus.89749

**Published:** 2025-08-10

**Authors:** Takashi Asahi, Shiro Horisawa, Takuto Nakamura, Hiroyuki Kajimoto, Ichiro Takumi

**Affiliations:** 1 Department of Neurosurgery, St. Marianna University School of Medicine, Kawasaki, JPN; 2 Department of Neurosurgery, Kanazawa Neurosurgery Hospital, Nonoichi, JPN; 3 Department of Neurosurgery, Tokyo Women’s Medical University, Tokyo, JPN; 4 D3 Center, University of Osaka Graduate School, Osaka, JPN; 5 Graduate School of Informatics and Engineering, The University of Electro-Communications, Tokyo, JPN

**Keywords:** hanger reflex, limb dystonia, yips, zolpidem, movement disorders

## Abstract

Dystonia is a movement disorder characterized by sustained or intermittent muscle contractions that result in abnormal, often repetitive movements, postures, or both. Zolpidem has been reported as an effective treatment for dystonia. We describe the case of a 24-year-old male long-distance runner with dystonia who successfully resumed competitive running by using a combination of a hanger reflex-inducing device and treatment with zolpidem. The patient presented with a sensation of weakness in his right foot while running. Video analysis of the running gait confirmed internal to external rotation of the right lower limb. At treatment initiation, a wrist-type hanger reflex induction device was adapted for use on the right ankle. At three months, the patient was able to run up to 20 km without any symptoms. He was subsequently prescribed zolpidem, which ultimately helped him to complete a full marathon without experiencing any symptoms. This report suggests that the combination of zolpidem and a hanger reflex device may be a promising, noninvasive treatment option for runner’s dystonia.

## Introduction

Dystonia is a movement disorder characterized by sustained or intermittent muscle contractions that cause abnormal, often repetitive movements, postures, or both [1]. It can affect any body part, including the feet. The onset of foot dystonia can be a severe problem for long-distance runners, often forcing them to abandon their flourishing careers [2,3]. The hanger reflex is a phenomenon that induces a sensation of head rotation when a wire hanger is worn on the head. This effect has been observed in various body parts [4-9] and has shown efficacy in the treatment of cervical and wrist dystonia [6]. Recently, zolpidem has been reported to be effective in treating dystonia [10-12]. In this report, we discuss the case of a runner with dystonia who successfully resumed his competitive athletic activities using a combination of a hanger reflex induction device and treatment with zolpidem.

## Case presentation

A 24-year-old male, a long-distance runner and member of a corporate track and field team with no significant medical or family history, presented with a sensation of weakness in his right foot while running, which he had been experiencing for two to three years. When increasing speed during long-distance runs, he became aware that his right leg turned from inside to outside on its own. The symptoms had initially appeared after he had changed his running shoes, but had persisted even after he had reverted to his original footwear. This abnormal movement had resulted in unstable running. He visited our hospital several months after the onset of symptoms.

**Figure 1:**
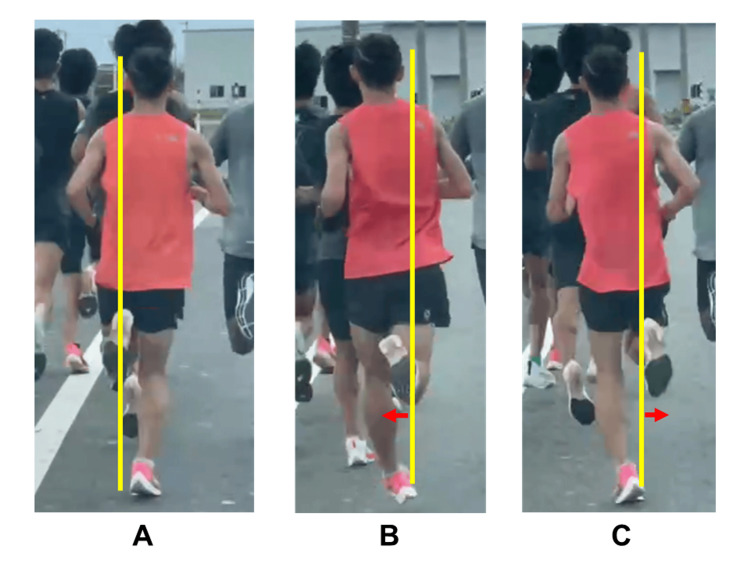
Pretreatment posterior view photographs of the patient while running A: When the left foot is lifted, it rises straight upward. B: When the right foot is lifted, it moves inward. C: After rotating outward, the right foot makes contact with the ground

A video analysis of the running gait confirmed internal-to-external rotation of the right lower limb (Figure 1, Video 1). After landing, the right foot first rotated inward and then outward before landing. He had no symptoms except while running. Given the involuntary, stereotyped, and task-specific movements, he was diagnosed with runner's dystonia.

**Video 1:** Posterior view video while running (~41s) The patient is able to run, but the right foot turns inward during the upswing and rotates outward upon landing. (41s ~) Demonstration of the device application method. When the device was worn to induce the hanger reflex, it appeared in the direction of internal rotation; foot rotation during running was inhibited

At treatment initiation, a wrist-type hanger reflex induction device was adapted for use on the right ankle (Video 1) [6,8]. The device was adjusted to induce an internal rotation reflex. When adjusted to induce the opposite rotation, no improvement in the symptoms was observed. The device was worn only during running practice. Practice was performed almost every day. Following the initiation of treatment, the distance the patient could run gradually increased. After three months, the patient was able to run up to 20 km without any symptoms. Although he was aware that the device was effective in improving his symptoms, he noticed blisters on the Achilles tendon area where the brace was in contact with his tendon after wearing it for a long time. Then, treatment with zolpidem, which has been reported to be effective for dystonia, was started.

Zolpidem was administered before all practice sessions and competitions. The patient initially took 10 mg of zolpidem, but given his concerns about its side effects, the dose was reduced to 7.5 mg, and he was able to continue the treatment with efficacy obtained. The patient took 7.5 mg of zolpidem before competitions, enabling him to participate in the races. After seven months, the use of the device became unnecessary. One year after the initiation of treatment, the patient returned to full competition using only pre-competition zolpidem. At 15 months post-initiation of treatment, the patient competed in a full marathon, finishing in two hours and 28 minutes, and was placed among the top positions. This confirmed the complete return to competitive running. After three years of treatment, he no longer experiences symptoms during normal practice, even though he consumes zolpidem as a precautionary measure before competitions.

## Discussion

This report demonstrates the successful treatment of dystonia in a marathon runner using a combination of a hanger reflex device and zolpidem, which enabled the patient to return to competitive running. The hanger reflex device initially caused improvement with symptom reduction observed only when the device was adjusted to induce internal rotation, suggesting that there is a specific hanger reflex effect rather than compression only. The initiation of pre-competition zolpidem enabled a complete return to competitive running. Runner's dystonia, known as "Nukenuke disease" in Japan, can be career-ending for long-distance runners given the lack of established treatments. A report of 13 cases of runner's dystonia showed that treatments including botulinum toxin, levodopa, clonazepam, trihexyphenidyl, and physical therapy had limited efficacy, with all patients retaining significant symptoms [2]. Although global pallidus internus deep-brain stimulation has shown promise, its application is limited in young athletes, given its invasive nature [3]. Ventro-oral thalamotomy has also been reported to be effective for exercise-induced lower limb dystonia; however, it is also invasive [13,14].

The hanger reflex is considered to be an illusion induced by skin shear force [4]. When a wire hanger is placed over the head and the long edge of the hanger attaches to the anterior temporal region, the skin of the anterior temporal region shifts outward, causing the head to rotate. The hanger device for the head is oval in shape. It is manually rotated, and when the hand is released, the anterior temporal skin is sheared in the opposite direction, causing the head to rotate in the sheared direction. The device for the wrist is similarly oval and, when worn at an angle to the foot bone, produces a hanger reflex in the direction of the skin displacement. Miyakami et al. experimentally demonstrated that the hanger reflex can appear simply by shifting the facial skin laterally [4]. When tape was applied to the frontal area and cheeks of the subjects and pulled with a rubber band, a positive correlation was found between the force of skin pulling and the angle at which the head turned. The hanger reflex was shown to be an illusory force sensation. We reported that it occurs in 85.4% of Japanese healthy individuals [15]. This phenomenon was first reported in 1991 when head movement was found to be suppressed in patients with spasmodic torticollis by wearing a square cardboard box on the head [16]. Asahi et al. developed and clinically applied a hanger reflex induction device [6,17].

The hanger reflex has been confirmed to be induced not only in head rotation but also in various body parts, including the anteroposterior direction of the head, wrists, elbows, shoulders, and lower limbs [4-9,18]. Studies have also been conducted to induce the appearance of the hanger reflex in the lower extremities. A pneumatic balloon-based device to induce the hanger reflex in the leg was developed, and the amount of the hanger reflex appearance in the leg was quantified in the thigh, knee, lower leg, and ankle areas [19]. It was reported that the hanger reflex was elicited in each of these areas, but the knee area was the most likely to elicit the reflex. In our case, the device was applied so that the skin of the leg was shifted inward during application to induce the hanger reflex and produce the sensation of leg rotation. Similar to a report describing improvement in cervical dystonia, the abnormal neural circuits in the brain seemed to be overridden by the information from the cutaneous sensation. The effectiveness of the hanger reflex device in this patient suggests its potential as a novel treatment method for runner's dystonia.

The efficacy of zolpidem in dystonia has been previously reported, and its efficacy was confirmed in this patient [10-12]. Zolpidem, a widely used hypnotic agent, enhances GABA transmission by selectively activating the GABA-A receptor α1 subunit. A recent randomized, double-blind, placebo-controlled crossover trial examined the efficacy of a single dose of zolpidem for task-specific dystonia (e.g., writer’s cramp and musician's dystonia) [12]. It significantly reduced dystonia severity scores and led to an immediate improvement in task-specific dystonia. Furthermore, combined evaluation with transcranial magnetic stimulation and FDG-PET suggested that excitability suppression in the primary motor cortex and functional changes in higher neural networks involved in sensory-motor integration (left inferior parietal lobule, cingulate gyrus, and cerebellum) may be involved [12]. The side effects of the drug may include sleepiness; a study of zolpidem administration for musician's cramp reported the side effect of sleepiness [11]. In a report involving a patient with residual postoperative dystonia symptoms, the dosage was adjusted to 2.5-10 mg per dose [10]. In our case, the 7.5 mg dosage was found to be effective without causing any side effects. Importantly, zolpidem is not included on the World Anti-Doping Agency prohibited list, making it suitable for use by competitive athletes [20].

## Conclusions

This report suggests that the combination of a hanger reflex device and zolpidem may offer a promising, noninvasive treatment option for runner’s dystonia. Since there is no current established treatment for runner's dystonia, this method is a new nonsurgical treatment option. However, as this is a single case report, further studies and additional case reports are warranted to validate the efficacy and generalizability of this method.
